# Editorial: *Bifidobacteria*: exploring the roles of these microbiome guardians and their effects on human health

**DOI:** 10.3389/fmicb.2026.1861676

**Published:** 2026-05-20

**Authors:** Mitesh Patel, Grace Nkechinyere Ijoma, Malgorzata Ziarno

**Affiliations:** 1 Department of Bioinformatics, Faculty of Engineering and Technology, Marwadi University, Rajkot, Gujarat, India; 2 Department of Environmental Sciences, College of Agricultural and Environmental Sciences, University of South Africa, Florida, Roodepoort, South Africa; 3 Department of Food Technology and Assessment, Institute of Food Science, Warsaw University of Life Sciences—SGGW (WULS—SGGW), Warsaw, Poland

**Keywords:** gut-associated lymphoid tissues (GALT), gut-brain axis, immune activation, metabolic diagnosis, microbial diversity, probiotics

The human microbiome is increasingly recognized as a dynamic, interactive ecosystem that shapes health across the lifespan. Among its many inhabitants, *Bifidobacteria* stand out as early colonizers and long-term contributors to immune development, metabolic balance and microbial homeostasis. Yet emerging research continues to expand our understanding of their influence, revealing roles that extend far beyond the gut and into systemic physiology. This Research Topic brings together 18 contributions that collectively deepen our appreciation of *Bifidobacteria* as active regulators of human health.

Several studies in this Research Topic highlight how specific *Bifidobacterium* strains may influence disease pathways in terms of modulation and immune regulation. Experimental work exploring glioma models, for example, suggests that certain *Bifidobacterium* species can modulate tumor-related signaling pathways and reshape both gut and tumor microbiota, offering intriguing avenues for future therapeutic exploration (Fan et al.). While preclinical, these findings reinforce the broader concept that microbial interventions may complement conventional oncology.

Other contributions focus on inflammatory and autoimmune conditions. Research on very early-onset inflammatory bowel disease associated with IL10RA mutations reports reduced microbial diversity and diminished *Bifidobacterium* abundance, while supplementation with *B. breve* showed protective effects in experimental settings (Xu et al.). Complementing this, studies on *B. adolescentis* and *B. lactis* V9 describe strain-specific capacities to modulate inflammation, support barrier integrity and influence immune pathways, underscoring the therapeutic potential of targeted probiotic strategies (Li et al.; Duan et al.).

A recurring theme across several papers is the metabolic versatility of *Bifidobacteria* and their relevance to host physiology in terms of metabolic health, obesity and systemic interactions. Work on *B. pseudocatenulatum* demonstrates strain-level differences in the utilization of plant-derived glycans, highlighting the ecological adaptability of this species within the gut environment (Sanchez-Gallardo et al.). This metabolic flexibility may help explain its repeated association with improved metabolic outcomes, including reduced lipid accumulation in obesity-related models (Wu et al.).

Associative studies examining obesity and systemic lupus erythematosus further suggest that *Bifidobacterium* abundance may interact with inflammatory and coagulation markers, although these relationships appear strongly modulated by host factors such as BMI (Chero-Sandoval et al.). A meta-analysis included in this Topic adds nuance, reporting modest but consistent improvements in body weight and insulin regulation following *Bifidobacterium* supplementation (Huang and Cheng).

Beyond metabolic health, emerging axes such as the gut-thyroid and gut-vascular pathways are explored. Depletion of *Bifidobacterium* was linked to altered iodine metabolism and goiter development in experimental models (Liao et al.), while a scoping review highlights how microbial metabolites may influence vascular inflammation and atherosclerosis progression (Zhang et al.).

The systemic reach of *Bifidobacteria* is further illustrated in studies examining neurobiology and stress regulation. They emphasize the role of *Bifidobacteria* with regards to microbial metabolites production, neurobiology, and host signaling, A review on the microbiota–depression interface highlights how microbial metabolites influence mitochondrial function, neurotransmitter balance, and immune signaling, offering mechanistic insights into microbiome-based mental health interventions (Zhao et al.). In children, associations between *Bifidobacterium* abundance and DNA methylation of stress-related genes suggest potential microbiome–epigenetic interactions relevant to hypothalamic-pituitary-adrenal axis regulation (Harker et al.).

Several contributions expand our understanding of *Bifidobacteria* at the genomic and molecular levels with emphasis to genomic diversity, ecological interactions and functional molecules. Genomic analyses of *B. dentium* reveal its ability to utilize both dietary glycans and human milk oligosaccharides, challenging traditional ecological assumptions about this species (Gonzaga et al.). Reviews of *B. animalis* subsp. *lactis* BB-12™ consolidate decades of evidence supporting its probiotic functions, while work on teichoic acids highlights their importance in adhesion and immune modulation (Collins et al.; Longhi et al.).

Finally, studies examining interactions between *Bifidobacterium* and *Akkermansia muciniphila* demonstrate how dietary glycans shape interspecies cooperation, influencing protein expression and gut barrier function (Sulaiman et al.). These findings reinforce the idea that *Bifidobacteria* operate not in isolation but within a complex microbial network.

Taken together, the contributions in this Research Topic highlight three overarching insights. First, strain-specific functional diversity is central to understanding the metabolic and therapeutic potential of *Bifidobacteria*. Second, these microorganisms play a pivotal role in maintaining intestinal barrier integrity and immune homeostasis, particularly under pathological conditions. Third, their influence extends well beyond the gut, intersecting with systemic processes through emerging axes such as the gut–brain, gut–vascular, and gut–thyroid pathways.

Despite these advances, important challenges remain. Individual microbiome variability, differences in experimental models and the absence of standardized clinical protocols continue to limit translational progress. Addressing these gaps will require integrative approaches that combine multi-omics technologies, artificial intelligence and rigorously designed clinical trials.

**Figure 1:**
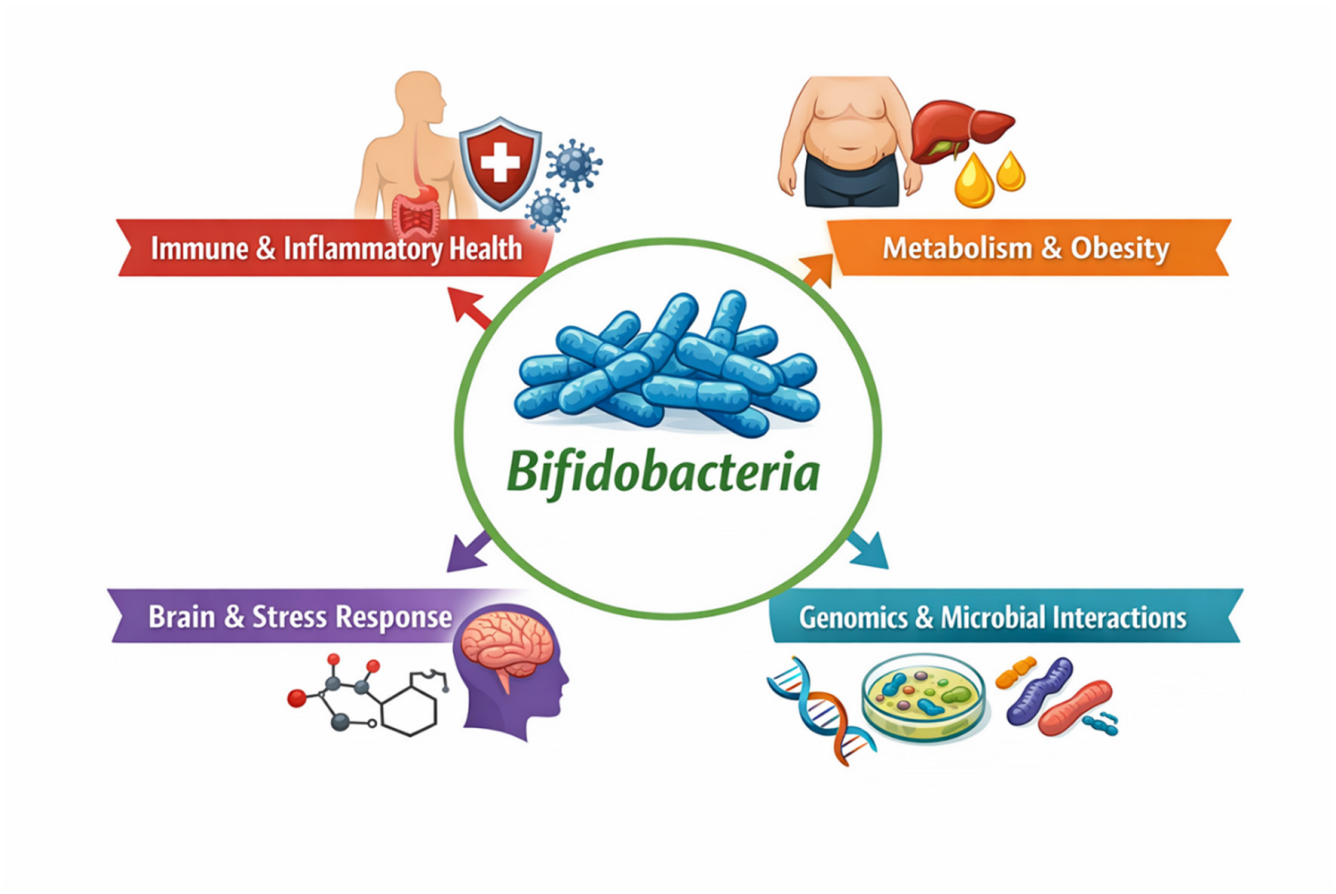
Interconnected roles of *Bifidobacteria* in systemic and gut health.

This Research Topic offers a comprehensive and forward-looking perspective on the diverse roles of *Bifidobacteria* in human health. Far from being passive commensals, these microorganisms emerge as active regulators of physiological processes across multiple organ systems as shown in Figure 1. Continued research in this field promises to accelerate the development of next-generation microbiome-based therapies capable of addressing complex and multifactorial diseases.

